# LncRNAs signatures associated with cuproptosis predict the prognosis of endometrial cancer

**DOI:** 10.3389/fgene.2023.1120089

**Published:** 2023-04-12

**Authors:** Shiyuan Qi, Huiyan Feng, Xiaomao Li

**Affiliations:** Department of Gynecology, The Third Affiliated Hospital of Sun Yat-sen University, Guangzhou, China

**Keywords:** cuproptosis, LncRNAs, endometrial cancer, targeted therapy, prognosis

## Abstract

**Background:** Endometrial cancer (UCEC) is the sixth most common cancer in women, and although surgery can provide a good prognosis for early-stage patients, the 5-year overall survival rate for women with metastatic disease is as low as 16%. Long non-coding RNAs (LncRNAs) are thought to play an important role in tumor progression. Cuproptosis is a recently discovered form of cell death in which copper binds directly to the lipoacylated component of the tricarboxylic acid (TCA) cycle. The aggregation of these copper-bound listed mitochondrial proteins and the loss of Fe-S cluster proteins trigger proteotoxic stress, which leads to cell death. Therefore, the aim of this work was to investigate the role of Cuproptosis-related LncRNAs signaling in clinical prognostic prediction and immunotherapy, as well as the relationship between tumor mutation burden.

**Methods:** Genomic, clinical and mutational data of endometrial cancer patients were presented in the TCGA database, and cuproptosis-related genes obtained from related studies. Coexpression analysis and Cox regression analysis were used to construct prognostic features. Patients were divided into high risk group and low risk group, and then ROC, survival rate, risk curve, principal component analysis, independent prognostic analysis and clinical subgroup model validation were performed to observe the prognostic value of characteristics. Subsequently, the GO and genomic KEGG enrichment and immune-related functions of LncRNAs as well as the tumor mutation burden were analyzed.

**Results:** In 548 UCEC case data, we identified five associated LncRNAs co-expressed with cuproptosis genes, and we found that high-risk patients had poorer overall survival (OS), progression-free survival (PFS), and higher mortality. Independent prognostic analysis, ROC showed that the LncRNAs associated with cuproptosis could accurately predict the prognosis of patients. Enrichment analysis revealed that the biological functions of LncRNAs were related to tumorigenesis. We also discovered suppression of immune-related functions in high-risk patients with oncogene mutations, higher tumor mutation burden in low-risk patients, and longer overall survival in patients with higher tumor mutation burden.

**Conclusion:** The identification of five LncRNAs associated with cuproptosis can accurately predict the prognosis of patients with endometrial cancer, and may provide a new perspective for clinical application and immunotherapy.

## 1 Introduction

### 1.1 Overview of endometrial cancer

Endometrial cancer (UCEC) is the sixth most common cancer in women. Despite the rapid development of medical technology over the years, there were an estimated 417367 new cases and 97370 deaths worldwide in 2020 (Sung et al., 2021). Although 67% of women with endometrial cancer are diagnosed with localized disease and the 5-year overall survival rate is about 95%, the 5-year overall survival rate for women with metastatic disease is as low as 16%. Even patients with early-stage disease still had poor survival after recurrence, with 5-year overall survival reduced to 55% for pelvic recurrence and 17% for extrapelvic recurrence (Bieńkiewicz et al., 2022). Patients with initially diagnosed advanced disease (stage III/IV) have a higher risk of recurrence and are more likely to present with extrapelvic metastases at recurrence (Connor and Rose, 2018). Thus, there is an urgent need for reliable predictive biomarkers to identify patients at high risk for disease recurrence.

### 1.2 Cuproptosis

A research paper titled Copper induces cell death by targeting lipoylated TCA cycle proteins published in Science by Tsvetkov et al. in March 2022 demonstrated that copper toxicity occurs by a mechanism distinct from all other known mechanisms that regulate cell death, including cellular Apoptosis, ferroptosis, apoptosis, and necrosis, and refers to this previously uncharacterized cell death mechanism as Cuproptosis. Cuproptosis refers to the direct binding of copper to the lipoacylated components of the tricarboxylic acid (TCA) cycle. The aggregation of these copper-bound lipoacylated mitochondrial proteins and the loss of Fe-S cluster proteins trigger proteotoxic stress, which leads to cell death. Copper is an important cofactor, and copper homeostasis is critical for various physiological processes. Dysregulation of intracellular copper bioavailability can induce oxidative stress and cytotoxic effects (Ge et al., 2022). Recent studies have shown that copper levels are significantly elevated in the serum and tumor tissues of cancer patients compared with healthy patients (Blockhuys et al., 2017) (Ishida et al., 2013). Dysregulation of copper homeostasis may trigger cytotoxicity, and alterations in intracellular copper levels may affect cancer development and progression. Therefore, the study of cuproptosis not only reveals a new cell death pathway, but also provides new ideas for anticancer therapy. It is important to study the role of cuproptosis in tumorigenesis and progression.

### 1.3 LncRNAs

Long non-coding RNAs (LncRNAs) are linear RNAs, typically 200 nucleotides long, that regulate gene expression at the post-transcriptional or transcriptional level. It is known that the aberrant expression of LncRNAs is closely related to the development of malignant tumors (He et al., 2019). LncRNAs are important in the progression of endometrial cancer (Li and Wan, 2018). It has been found that LncRNA LINC01468 expression is lower in endometrial cancer patients than in controls, which may indicate that it acts as a protective factor against this malignancy (Bieńkiewicz et al., 2022). LncRNA DSCAM-AS1 promotes the progression of endometrial cancer by regulating Mir-136-5p (Li et al., 2021). LncRNAs that play a role in tumor suppression in endometrial cancer include: FER1L4, GAS5, MEG3, RP11-395G23.3, LA16C −313D11.11, Xist, Linc ROR, MALAT1, HOTAIR, OVAL, SRA, *etc.*, (Guo et al., 2015) However, the mechanism of cuproptosis-associated LncRNAs in endometrial cancer is still unclear.

In this study, the Cancer Genome Atlas (TCGA) was used to obtain transcriptional and clinical data of endometrial cancer patients. Cox regression analysis based on (LASSO) was used to determine the key cuproptosis-related LncRNAs affecting the prognosis of endometrial cancer, and to construct a risk score model for prognosis prediction of endometrial cancer. We identified the key role of cuproptosis-related LncRNAs in UCEC progression and found that the use of our risk score model could accurately predict the prognosis of patients. Immunoassay and functional enrichment analysis were also performed to identify the potential cellular pathways regulated by LncRNAs.

## 2 Materials and methods

### 2.1 Data and information

In May 2020, we obtained the gene expression data and clinical information (including age, pathological grade, survival time, *etc.*) of 548 UCEC cases and 41 healthy samples from TCGA official website (Table 1). 19 genes related to cuproptosis were obtained from previous studies (NFE2L2 NLRP3 ATP7B ATP7A SLC31A1 FDX1 LIAS LIPT1 LIPT2 DLD DLAT PDHA1 PDHB MTF1 GLS CDKN2A DBT GCSH DLST)(Tsvetkov et al., 2022)

**TABLE 1:** Clinical characteristics of UCEC patients involved in the study.

Clinical characteristics	Classification	Amount
Stage	I	342 (62.4%)
	II	52 (9.49%)
	III	124 (22.6%)
	IV	30 (5.47%)
Age	<60	181 (33.0%)
	≥60	364 (66.4%)
	Unknown	3 (0.6%)
Pathological type	Endometrioid adenocarcinoma	411 (75%)
	Serous endometrial adenocarcinoma	22 (4.0%)
	Mixed serous and endometrial carcinoma	115 (21%)
Histological grade	G1	99 (18.1%)
	G2	122 (22.3%)
	G3	327 (59.7%)
Survival state	ALive	503 (91.8%)
	Death	45 (8.2%)
Pelvic lymph node enlargement	Yes	56 (10.2%)
	No	338 (61.8%)
	Unknown	154 (28.1%)
Para-aortic lymph node enlargement	Yes	29 (5.3%)
	No	291 (53.1%)
	Unknown	228 (41.6%)

### 2.2 Expression of cuproptosis-related genes in UCEC

Our bioinformatics analysis is based on “R” (version 4.2.0) software. Firstly, “limma” and “pheatmap” in “R" were used to obtain differentially expressed cuproptosis-related genes in UCEC and healthy samples, and heat maps were drawn (log_2_FC > 0; FDR< 0.05). Next, univariate COX regression analysis and survival analysis were performed using the “limma” and “survival” packages (*p* < 0.05), and draw the forest map and survival curve, so as to obtain the cuproptosis-related genes with the largest difference in expression between UCEC and healthy samples.

### 2.3 Acquisition of cuproptosis-related LncRNAs

Cuproptosis-related LncRNAs were identified by the “limma” package in “R” (|Pearson R| > 0.5, *p* < 0.001). R package “ggplot2” “ggalluvial” was used to map the sankey diagram. The prognostic significance of cuproptosis-related LncRNAs were obtained by using (LASSO) Cox regression analysis (*p* < 0.05).

### 2.4 Construction of risk score model

The patients were randomly divided into a training group and a test group. As a result, five LncRNAs associated with cuproptosis were screened and used to construct a prognostic risk model. The risk score of each patient was calculated by the following formula:

$$Risk\;Score=\sum_{i=1}^{n}\left(Coefi*xi\right)$$

where Coefi is the coefficient and xi is the fragment number per million transcripts (FPKM) value of prognostically significant cuproptosis-related LncRNAs. The R “limma”, “reshape2”, “Tidyverse", and “GGPLOT2” packages were used to obtain heat maps of the correlation between LncRNAs involved in model construction and cuproptosis-related genes. After obtaining the risk score for each patient, patients could be divided into low and high risk groups according to the median risk value.

We then performed Kaplan-Meier (KM) analysis and PFS analysis using the “SurvMiner” and “survival”packages to investigate the differences in OS and PFS between the high and low risk groups. The “SurvMiner","Survival“ and “RMS” packages were used to construct ROC curve to verify the accuracy of survival prediction.

### 2.5 Construct the nomogram

Nomograms were drawn to determine the survival rates of patients at 1, 3, and 5 years using the “survival”, “rms” and “regplot” packages. Calibration curves were plotted to show the difference between the predicted and actual outcomes of the nomogram.

### 2.6 Get principal component analysis (PCA) and enrichment analysis

PCA was constructed by using “limma” and “Scatterplot3D″ package to examine the distribution of patients with different risk scores. Gene Ontology (GO) and Kyoto Encyclopedia of Genes and Genomes (KEGG) enrichment analyses of cuproptosis-related LncRNAs were performed by “clusterProfiler” package, and *p* < 0.05 and *p* < 1 were considered statistically significant.

### 2.7 Immunorelated function analysis, tumor mutation burden analysis and drug sensitivity analysis

“limma” and “GSVA” packages were used to analyze the difference of immune-related function in patients with endometrial cancer. *p* < 0.05 was considered statistically significant, and “PHEATMAP” package was used for result visualization. “Maftools” package was used to compare the relationship between risk score and tumor mutation burden (TMB). The difference between TMB and patient survival was investigated using the “survival” package, and *p* < 0.05 was considered statistically significant. “Prophet”, “ggplot2″, and “ggpubr” packages were used to screen therapeutic drugs and observe drug sensitivity (*p* = 0.001).

### 2.8 Validation of Risk Score Model and TCGA molecular model

“Ggplot2″, “ggalluvial” and “tidyverse” packages were used to obtain the relationship between the high-risk group in this study and the currently known TCGA molecular typing, and further verify the accuracy of the Risk Score Model constructed in this study.

### 2.9 Statistical analysis

ROC curve analysis and Kaplan-Meier survival analysis were used to analyze the efficacy of R sofware in predicting survival outcomes (version 4.2.0). Te link between a prognostic classifer and survival outcomes, as wellas other clinical parameters, was investigated using a Cox proportional model. When the *p*-value was less than 0.05, the results were considered statistically signifcant.

## 3 Results

### 3.1 Expression of cuproptosis-related genes in UCEC

**FIGURE 1:**
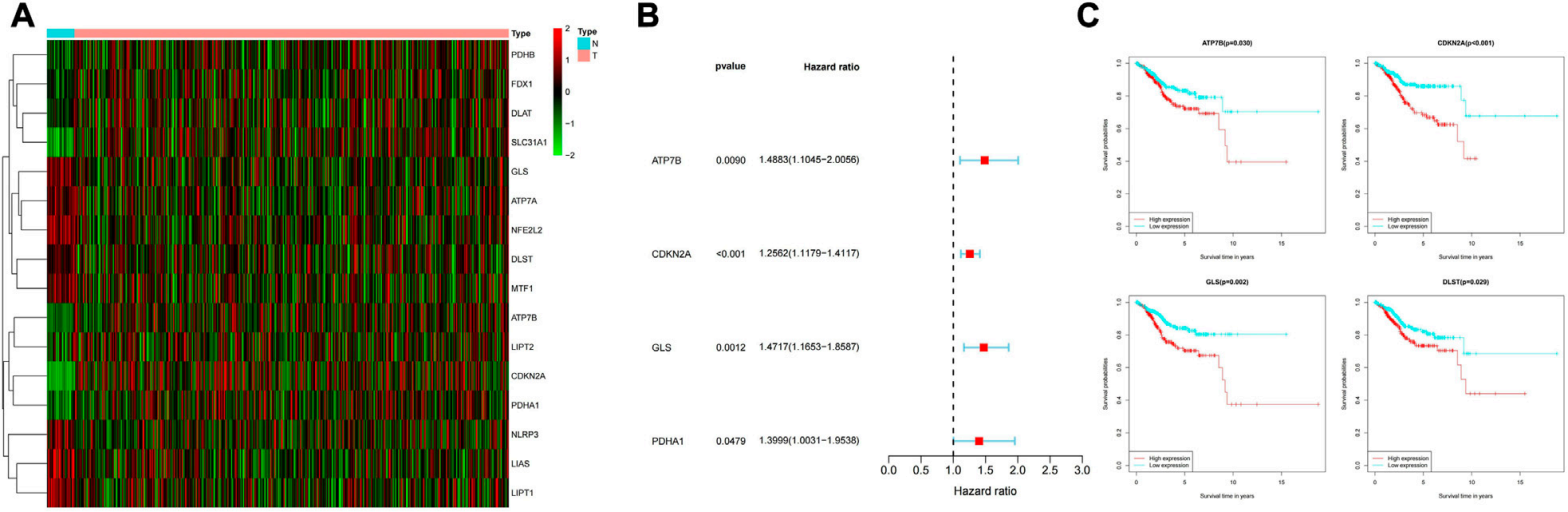
**(A)** Expression of cuproptosis-related genes in normal samples and samples from UCEC patients. **(B)** Normal samples and UCEC samples have differential expression of cuproptosis-related genes. **(C)** Relationship between expression of differentially expressed genes and survival possibility.

As shown in Figure 1, among the 19 cuproptosis genes, the expressions of ATP7B, CDKN2A, GLS and DLST in UCEC and normal tissues were different (*p* < 0.05), and the high expression of these four genes was also closely associated with poor prognosis.(Figure 1C).

### 3.2 Acquisition of cuproptosis-related LncRNAs and construction of risk prognostic model

**FIGURE 2:**
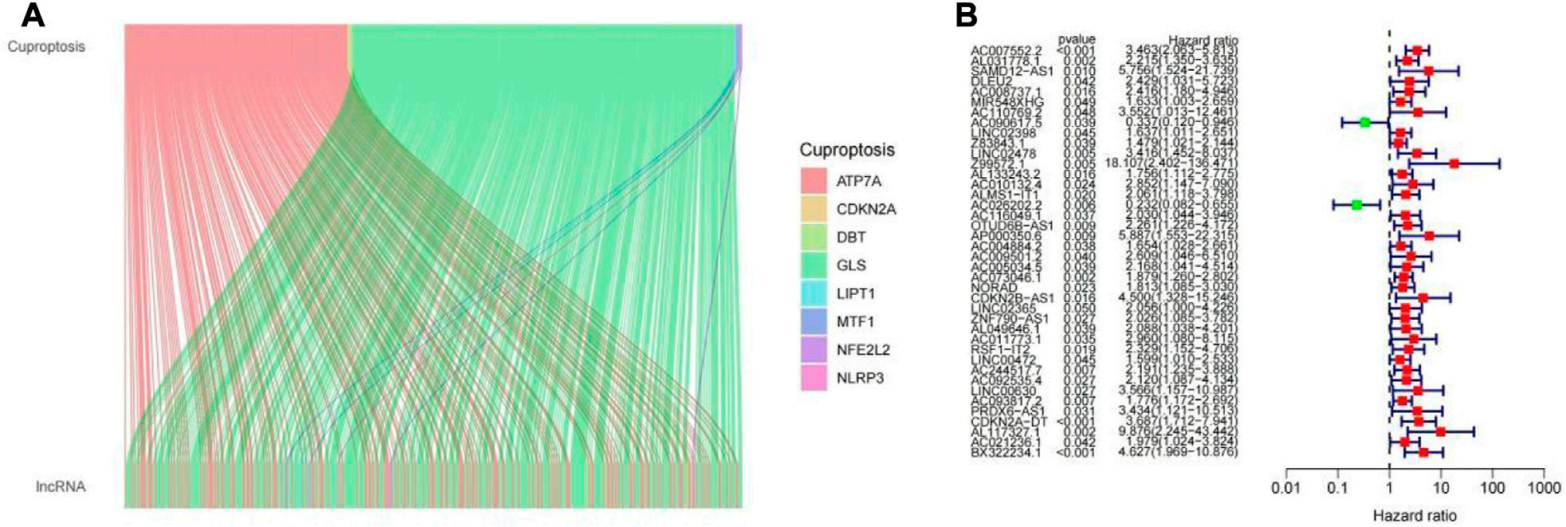
**(A)** LncRNAs associated with cuproptosis. **(B)** Difference values of LncRNAs analyzed by univariate COX regression in the training group.

**FIGURE 3:**
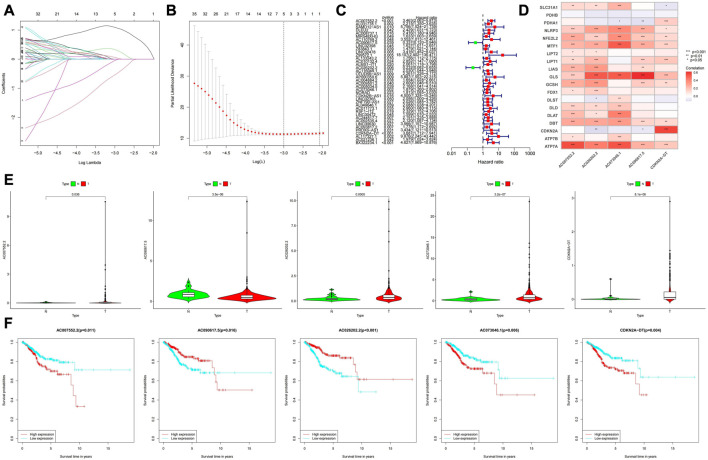
**(A,B)** LncRNAs screened by the LASSO-Cox regression model. **(C)** The green marks represent low-risk LncRNAs, and the red marks represent high-risk LncRNAs. **(D)** Relationship between cuproptosis-related genes and LncRNAs. **(E)** Expression levels of the 5 LncRNAs in tumor and normal tissues. **(F)** Prognostic value analysis of the 5 LncRNAs.

By the criteria of |R|>0.5 and *p* < 0.001, 467 cuproptosis-related LncRNAs were identified from 16,744 LncRNAs and 19 cuproptosis-related genes, and the related genes and cuproptosis-related LncRNAs were visualized using a Sankey diagram (Figure 2A). In training group, single variable COX regression analysis was used to identify the 40 cuproptosis-related LncRNAs, and forest map showed high and low risk of LncRNAs (Figure 2B). Lasso-cox regression analysis and cross-validation were used to identify five prognostic LncRNAs associated with cuproptosis (Figures 3A,B). Finally, multivariate COX analysis identified these five LncRNAs as independent prognostic factors. This study was also used to construct a prognostic risk prediction model. The risk score of this study = AC007552.2*1.56078256641192+(AC090617.5*-0.946605232564889)+(AC026202.2*-1.43809248679026) + (AC073046.1) *0.335343510163942) + ('CDKN2A-dt '*0.69728476). The correlation heatmap also showed the relationship between cuproptosis-related genes used to construct the prognostic model and LncRNAs (Figure 3D). We found that these five human LncRNAs were differentially expressed in tumor and normal tissues (Figure 3E), and they were closely related to the prognosis of patients (Figure 3F).

### 3.3 Survival analysis of the features

**FIGURE 4:**
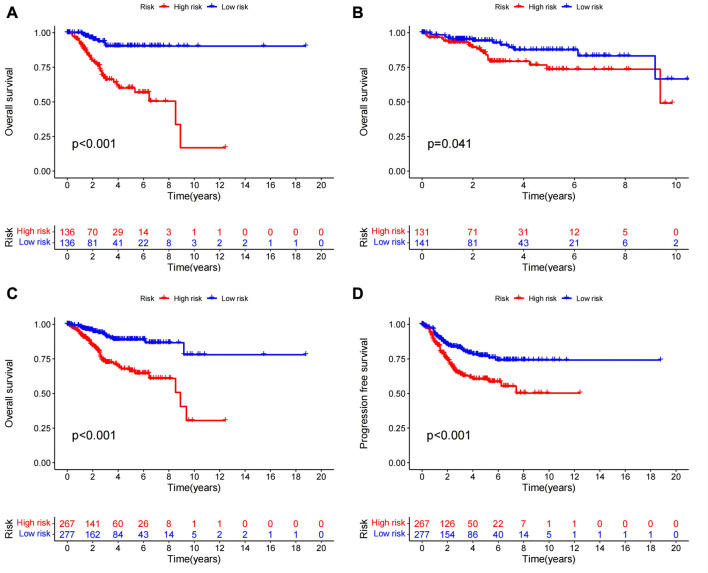
There were significant differences in survival analysis and PFS analysis between the high and low risk groups **(A)** KM analysis of the training group. **(B)** KM analysis of the test group. **(C)** The overall KM analysis. **(D)** PFS.

**FIGURE 5:**
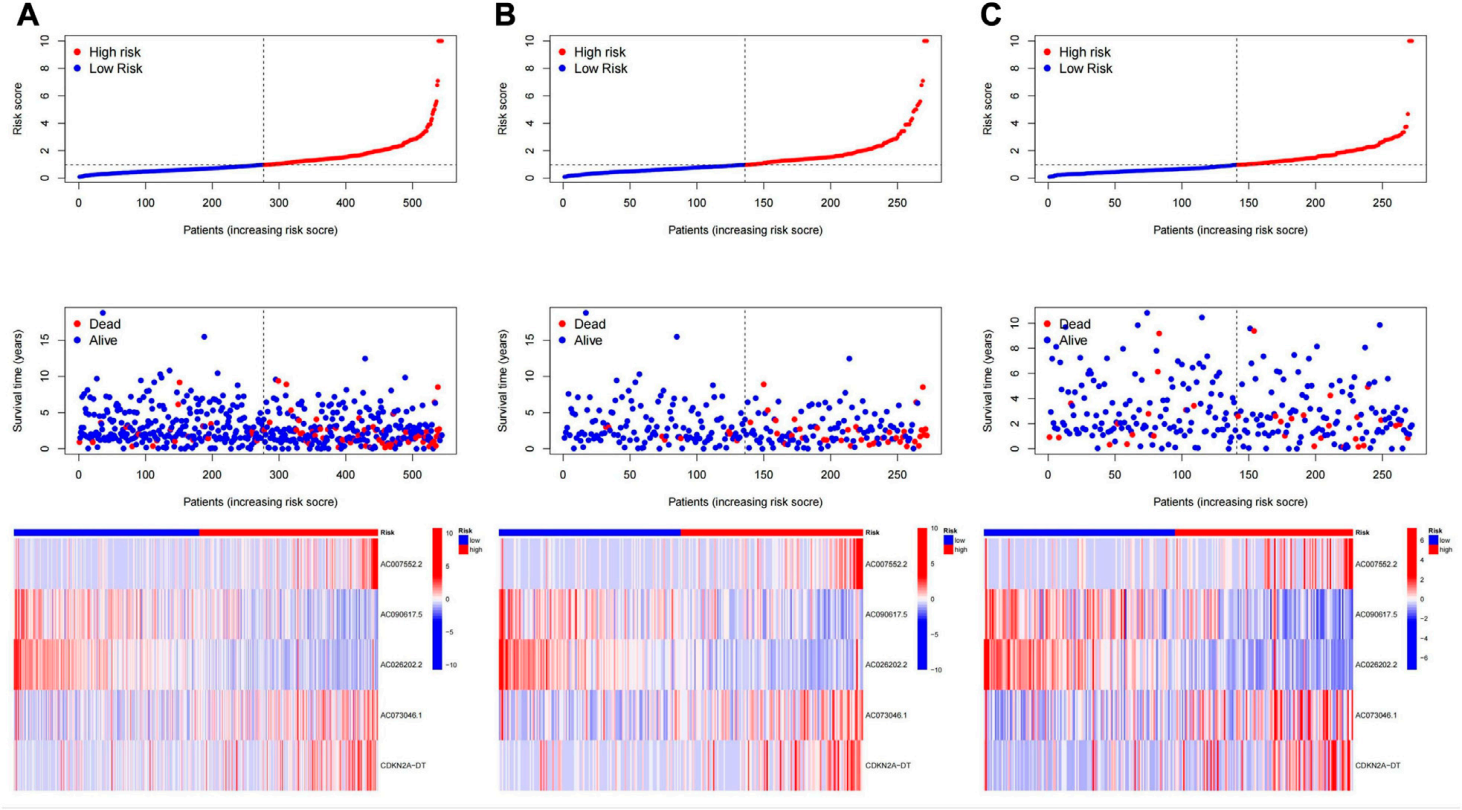
Risk plots for all samples **(A)**, training group **(B)**, and test group **(C)**.

**FIGURE 6:**
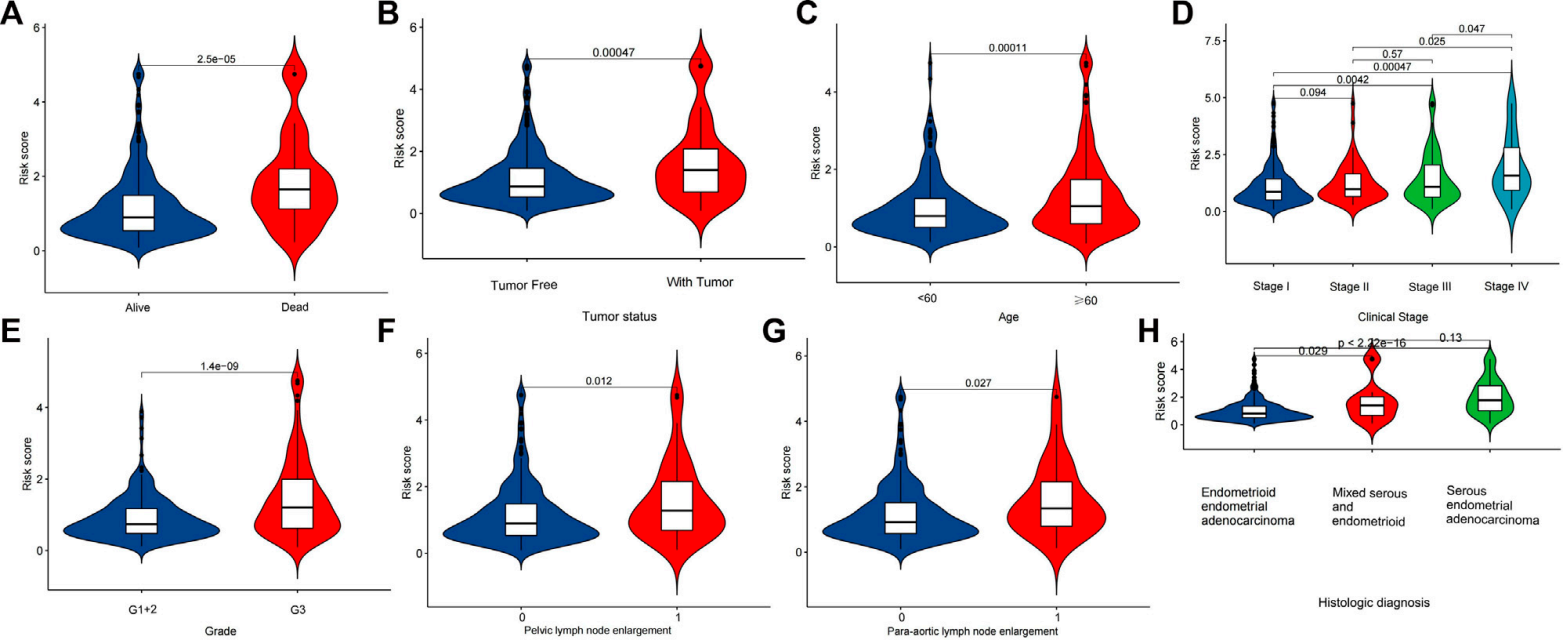
Relationship between Cuproptosis-related LncRNA signature and clinical characteristics. Analysis of diferences in risk scores between patients with diferent survival status **(A)**, recurrence status **(B)**, age **(C)**, stage **(D)**, grade **(E)**, pelvic lymph node enlargement **(F)**, para-aortic lymph node enlargement **(G)**, Histological diagnosis **(H)**.

The median value of the risk score was used as the cutoff value, and the patients were divided into low-risk group and high-risk group. We found that overall survival (OS) and progression-free survival (PFS) were significantly shorter in the high-risk group than in the low-risk group in the training, testing, and all groups (Figure 4). As shown in Figure 5, the risk curve reflected the relationship between risk score and survival status in UCEC patients, and we found that high-risk patients had a higher mortality rate than low-risk patients. The heat map showed the high risk and low risk levels of five LncRNAs. For example, AC007552.2, AC073046.1, CDKN2A−DT are high risk LncRNAs, and AC090617.5 and AC026202.2 are low risk LncRNAs. We also found high risk scores in patients with death (Figure 6A), recurrence (Figure 6B), age ≥60 years (Figure 6C), late clinical stage (Figure 6D), high histological grade (Figure 6E), pelvic and para-aortic lymph node enlargement (Figures 6F,G), and serous endometrial adenocarcinoma (Figure 6H), all of which indicate a poor prognosis in patients with higher risk scores.

### 3.4 Independent analysis of prognostic factors

**FIGURE 7:**
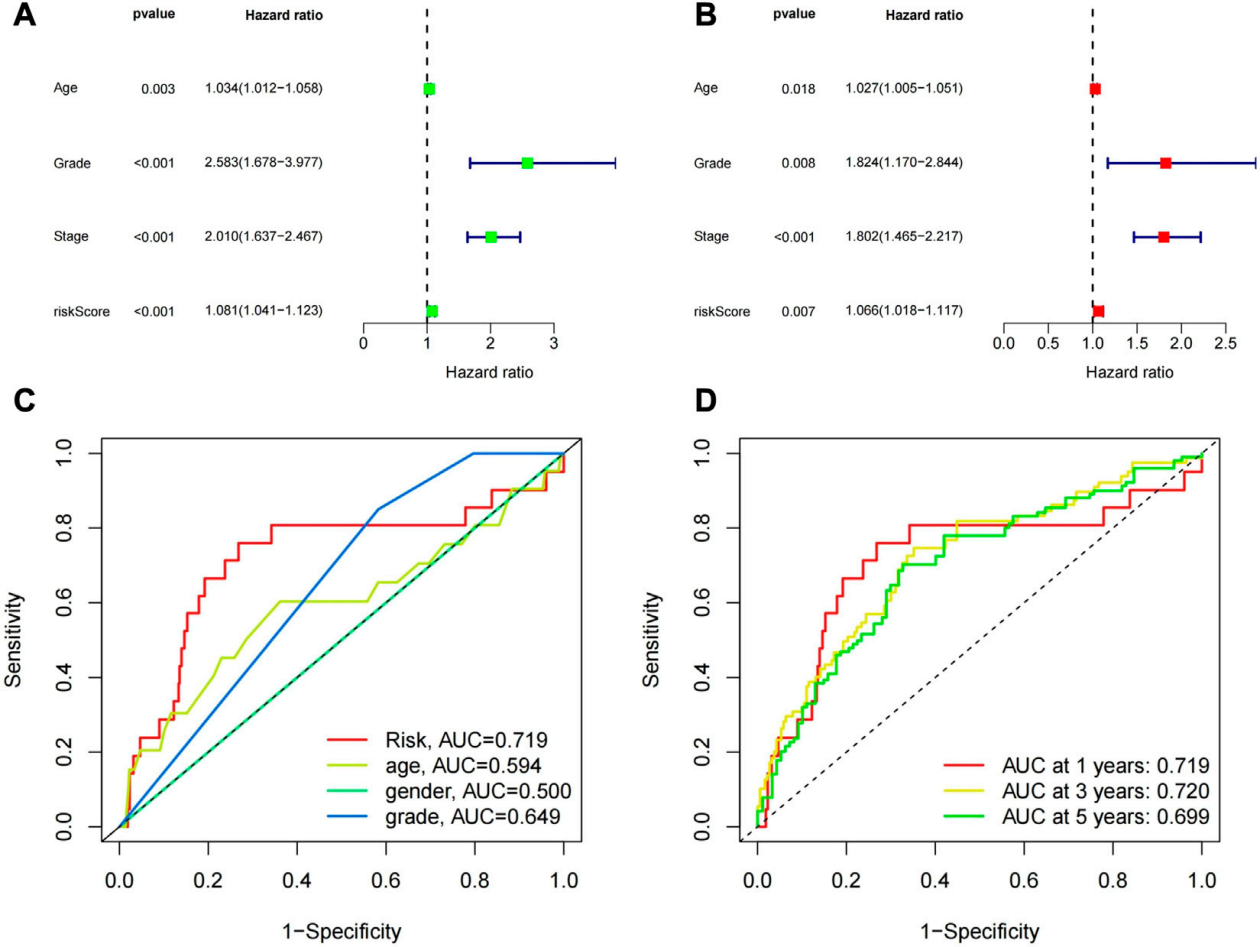
**(A)** Univariate regression analysis of the risk model and other clinical characteristics. **(B)** Multivariate regression analysis of the risk model and other clinical characteristics. **(C)**The ROC curve of the risk prediction model compared with other clinical factors. **(D)** The ROC curve of the risk prediction model at 1, 3, and 5 years.

Univariate and multivariate Cox regression analyses were used to determine whether the features we constructed could be used as independent prognostic factors, which are independent of other clinical characteristics (Figure 7A). Multivariate Cox regression analysis showed that age (HR = 1.024, 1.001 to 1.048, *p* = 0.037), pathological grade (HR 2.358, 1.531 to 3.630, *p* = 0.037), *p* < 0.001) and risk score (HR = 1.028, 1.016-1.040, *p* < 0.05) were independently associated with OS, indicating that prognostic characteristics were independent prognostic factors for UCEC patients (Figure 7B). Next, we used the receiver operating characteristic (ROC) curve to evaluate the predictive accuracy of the risk score. As shown in Figure 7C, the area under the ROC curve (AUC) of the risk score was 0.719, which was better than that of age (0.594) and pathological grade (0.649). Similarly, the area under the ROC curve (AUC) at 1, 3, and 5 years was 0.719, 0.720, and 0.699, respectively (Figure 7D), indicating that the prognostic features had good diagnostic significance.

### 3.5 Building a nomogram model

**FIGURE 8:**
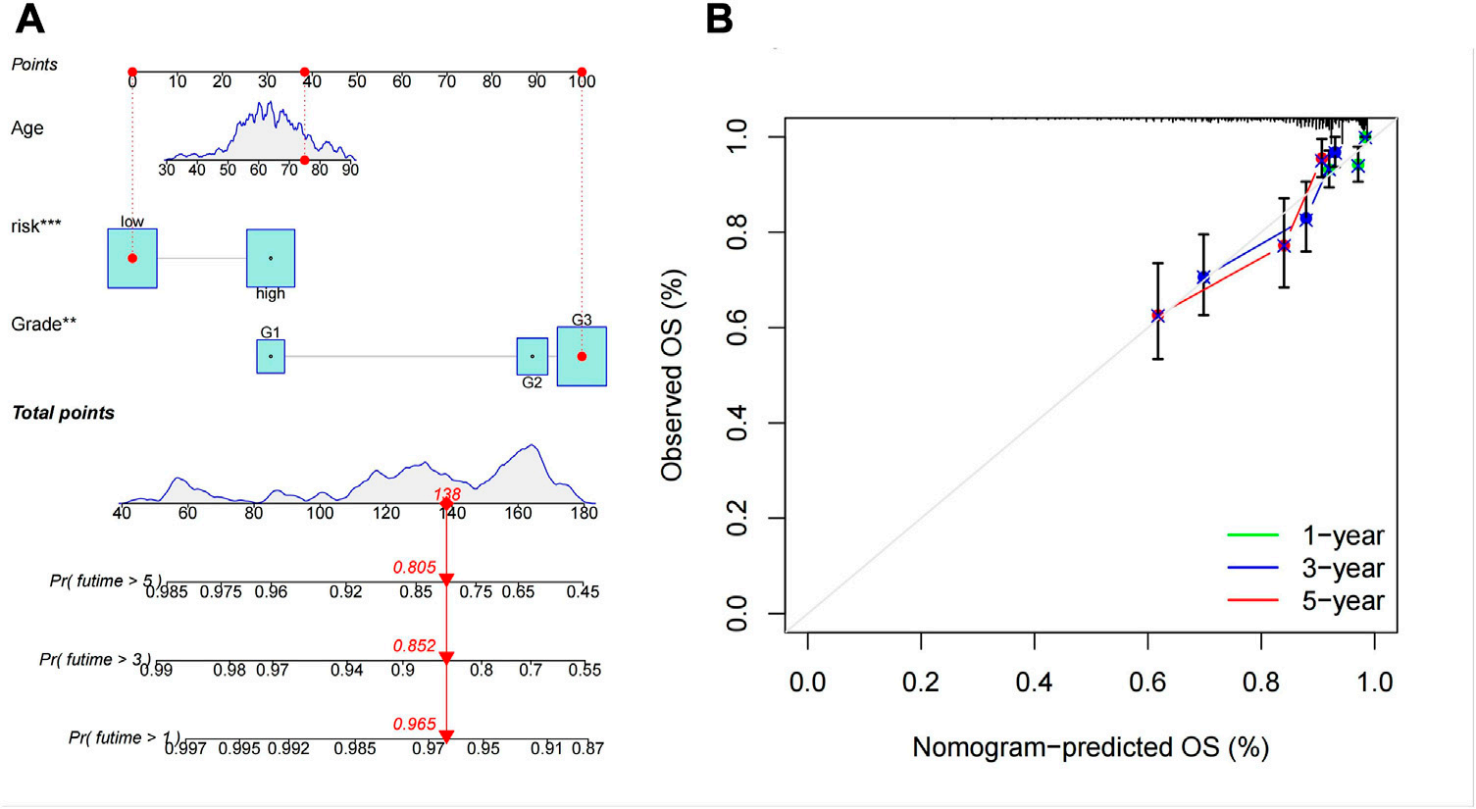
The predictive signifcance of the Cuproptosis-associated LncRNA signature was verifed in the nomogram model. **(A)** Nomogram combining the five Cuproptosis-associated LncRNA signatures. **(B)** Calibration plots of 1-, 3-, and 5-year survival probabilities.

We created a predictive nomogram based on three prognostic factors, including risk score, age, tumor tissue grade to directly predict the overall survival of UCEC patients (Figure 8A). It’s easy to know the probabilities of 1, 3, and 5 years OS by calculating total points. Finally, the calibration curve showed that the predicted probability was extremely consistent with the actual probability (Figure 8B).

### 3.6 Principal component analysis and enrichment analysis

**FIGURE 9:**
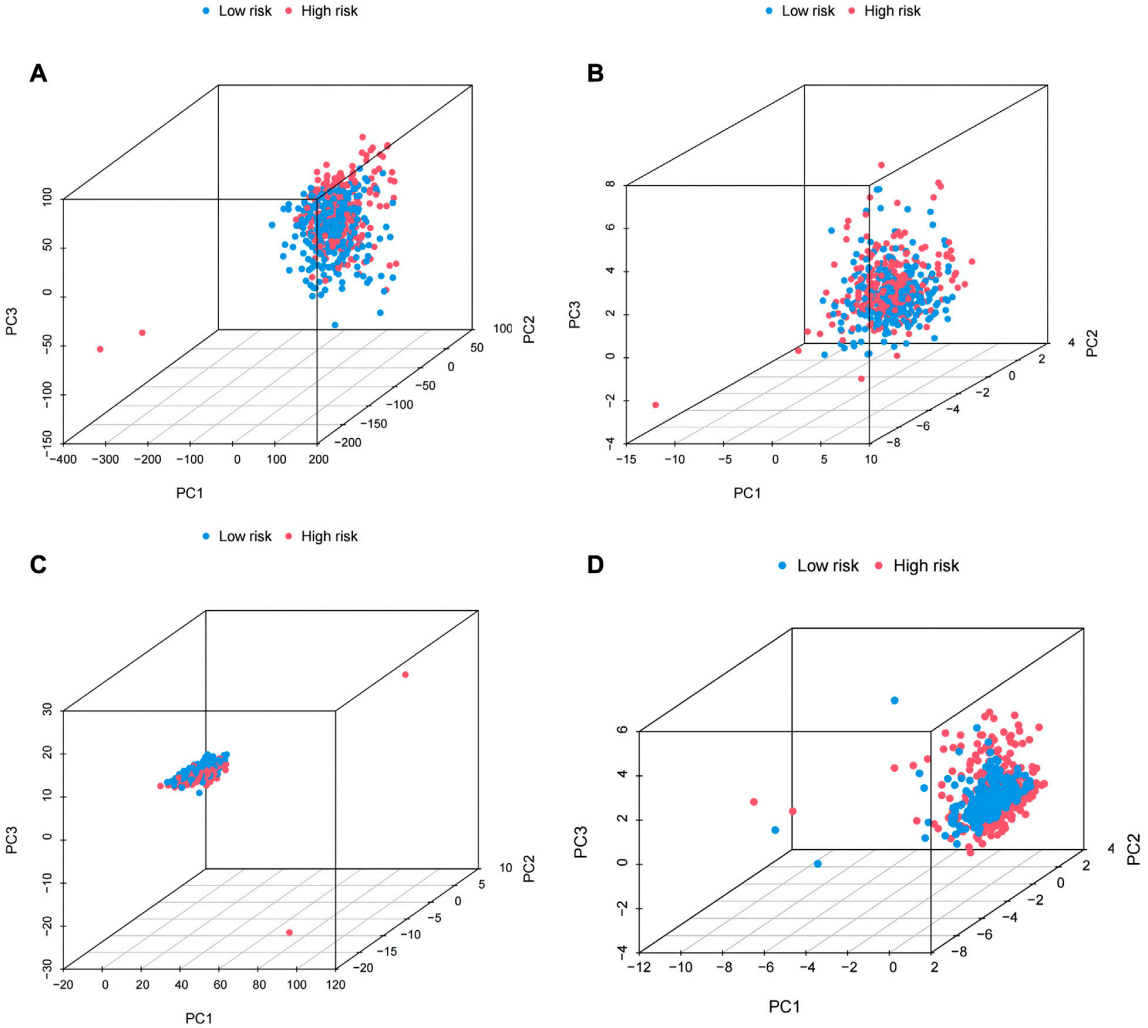
PCA analysis. **(A)** Plot for all genes. **(B)** Cuproptosis-related genes.**(C)** Cuproptosis-related LncRNA. **(D)** The LncRNA at risk.

**FIGURE 10:**
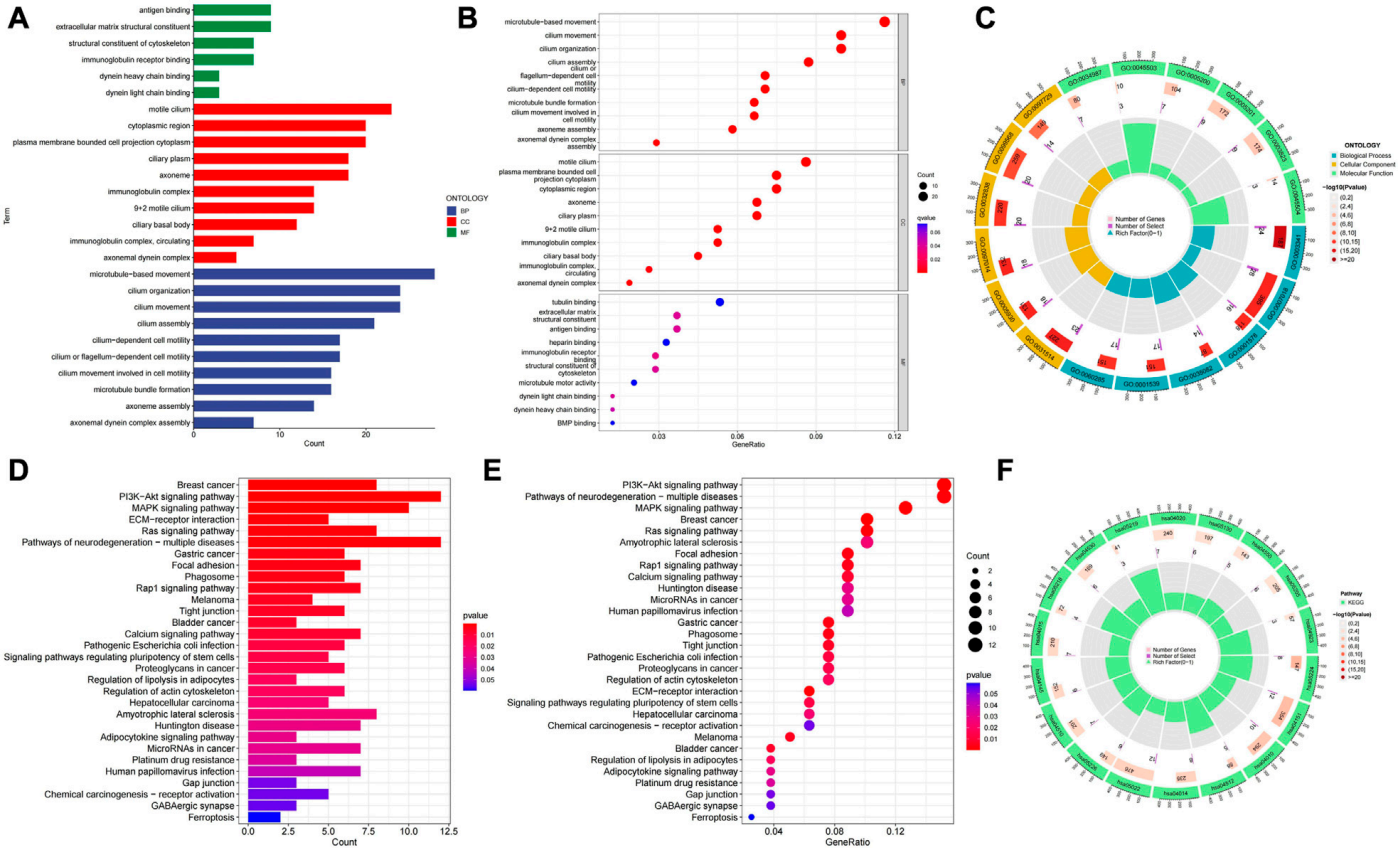
**(A–C)** GO enrichment analysis. **(D–F)** KEGG enrichment analysis.

We performed principal component analysis (PCA) to observe the distribution of all genes, cuproptosis-related genes, cuproptosis-related LncRNAs and risk LncRNAs in patients, and the results showed a clear distribution of risk LncRNAs, which proved that these LncRNAs were credible to be used to construct the prognostic model (Figure 9). GO results showed that the molecular function of cuproptosis-related LncRNAs was based on the movement of microtubules, and the biological process was more concentrated in antigen binding, while in cellular localization, it was mostly located in the cytoplasmic region (Figure 10A). KEGG analysis showed that these LncRNAs may be related to the PI3K-AKT signaling pathway, MAPK signaling pathway and a variety of neurodegenerative pathways, indicating that these LncRNAs are involved in the process of tumor development (Figure 10B).

### 3.7 Immunorelated function analysis, tumor mutation burden analysis and drug sensitivity analysis

**FIGURE 11:**
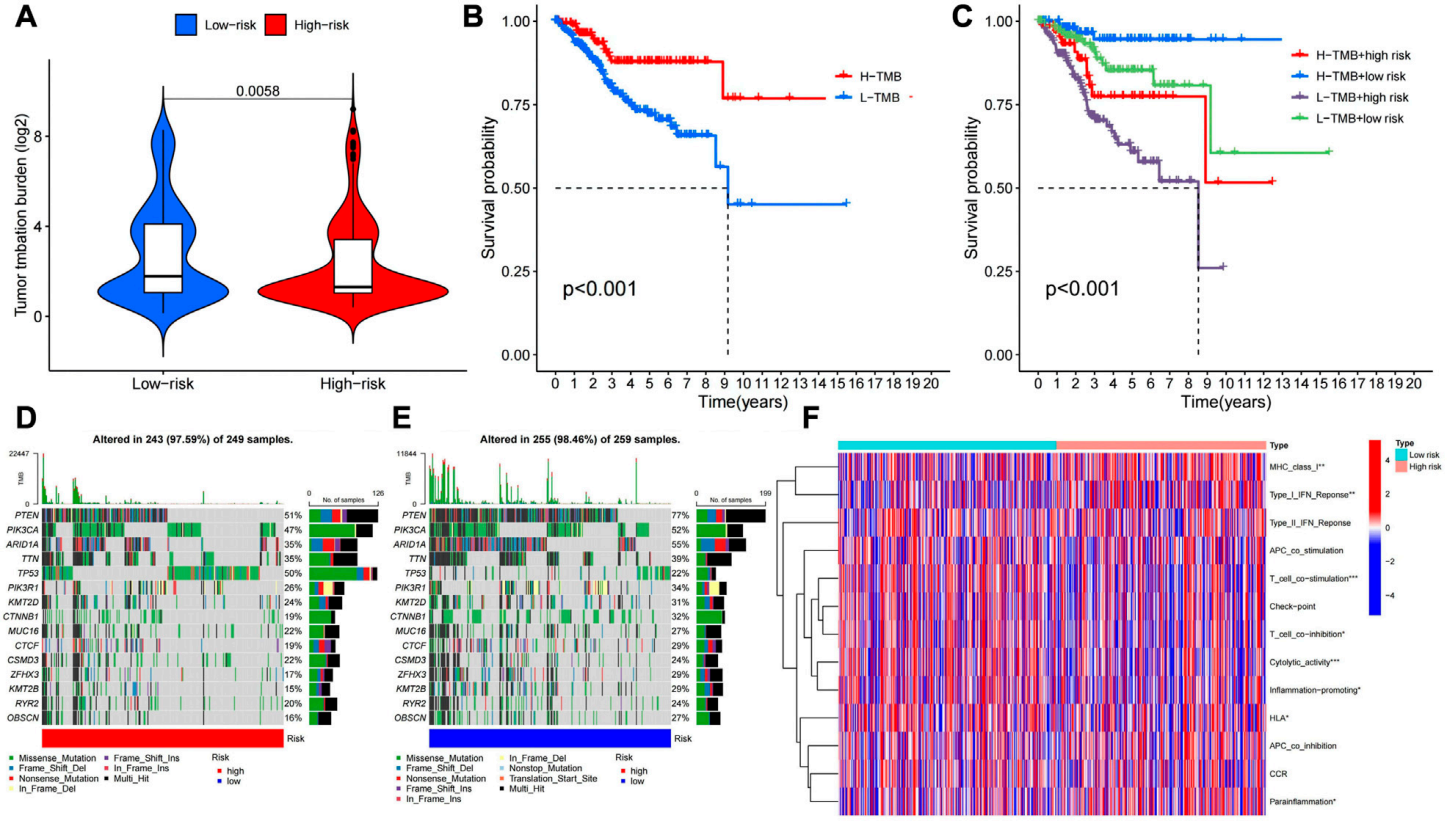
**(A)** TMB analysis of low and low risk groups. **(B)** Survival for high and low tumor mutation burden. **(C)**Survival rates for different tumor mutation burden combined with high and low risk. (d **(E)** Comparison of gene mutations in high and low risk groups. **(F)** Analysis of immune function in high and low risk groups.

**FIGURE 12:**
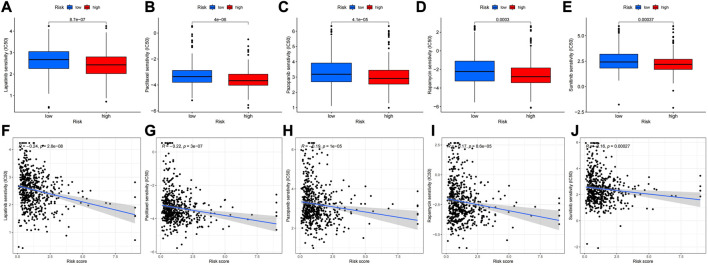
Drug sensitivity analysis. **(A–E)** Ic50 (the half maximal inhibitory concentration) values of different drugs (Lapatinib, Paclitaxel, Pazopanib, Rapamycin, Sunitinib) in the high-risk and low-risk groups. **(F–J)** Correlation between drug sensitivity and value at risk.

The R “limma” package was used to calculate the tumor mutation burden of the low and high risk groups, and it was found that the tumor mutation burden of the low risk group was significantly different from the high risk group (*p* < 0.05), and the tumor mutation burden of the low risk group was higher than that of the high risk group (Figure 11A) Then, we performed survival analysis of the samples again by using the “survival” and “SurvMiner” packages, and the results showed that there was a difference in the survival of the high and low mutation burden group (*p* < 0.001), and the survival rate of the high mutation burden group was higher. The comprehensive survival analysis combined with risk score showed that the survival time of the four groups was significantly different. The survival rate of patients with high mutation burden and low risk was the highest, while the survival rate of patients with low mutation burden and high risk was the lowest (Figure 11B, C) The waterfall plot revealed that the frequency of mutations in the high and low risk groups varies across genes, and for most genes, the frequency of mutations in the low risk group is higher (Figure 11D, E). Heat map of immune function analysis showed that T-cell costimulatory and cytolytic activity differed between the low and high risk groups (*p* < 0.001), and both were more active in the low risk group. (Figure 11F). Finally, we used the "PROPHECY" package to screen potentially effective antitumor agents, including Lapatinib, Paclitaxel, Pazopanib, Rapamycin, Sunitinib. We further analyzed the sensitivity of these drugs and found that patients in the high-risk group had lower IC50 values, indicating that patients in the high-risk group were more sensitive to these drugs (Figures 12A–J).

### 3.8 Validation of Risk Score Model and TCGA molecular model

**FIGURE 13:**
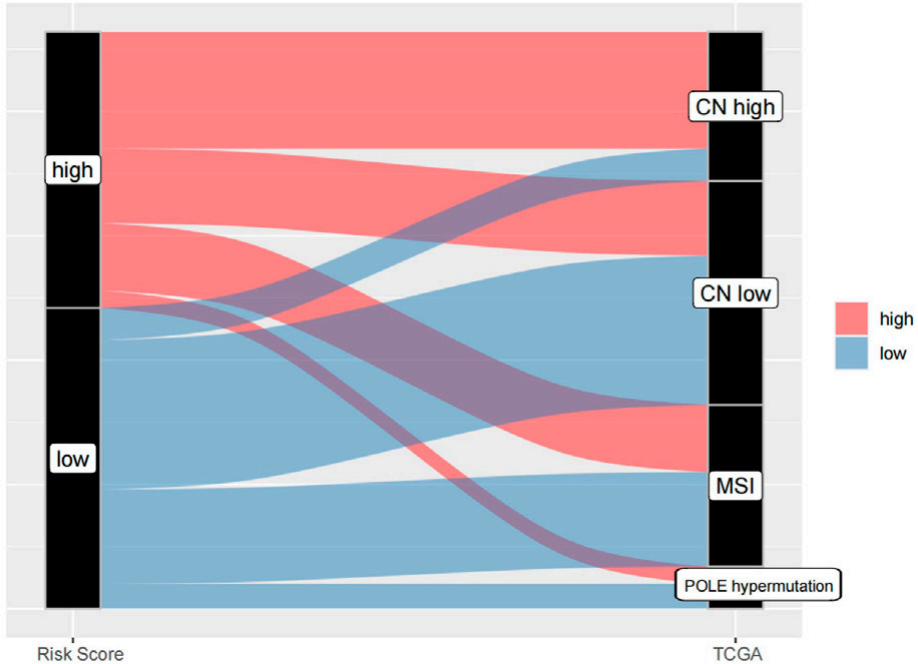
Validation of Risk Score Model and TCGA molecular model.

According to the Sankey chart (Figure 13), patients in the high-risk group were mostly concentrated in the CNH type, while patients in the low-risk group were mostly concentrated in the POLE hypermutation type, MSI type and CNL type.

## 4 Discussion

Although endometrial cancer most often presents early and has a good prognosis, there are still many women who will have a recurrence and cause death. Unfortunately, we have not improved survival outcomes for women with endometrial cancer. Therefore, the discovery of specific biomarkers for predicting the prognosis of endometrial cancer is of great importance. LncRNAs are emerging regulators involved in gene expression and a variety of physiological and pathological processes (Ransohoff et al., 2017; Li et al., 2020). Accumulating evidence suggests that LncRNAs play complex and precise regulatory roles in the occurrence and development of cancer by acting as oncogenes or tumor suppressors (Kim et al., 2018; Esposito et al., 2019). Cuproptosis is a new type of cell death, which is different from other known cell death modes, such as apoptosis and pyroptosis, but similar to zinc death and ferroptosis. Recently, many studies have used LncRNAs to predict the prognosis of endometrial cancer, but few studies have explored the co-regulatory role of cuproptosis-related LncRNA in endometrial cancer.

In our study, cuproptosis-related LncRNAs was obtained through the co-expression of LncRNAs and cuproptosis-related genes, and 5 cuproptosis-related LncRNAs with prognostic significance were obtained by univariate and multivariate Cox regression, including: AC007552.2, AC090617.5, AC026202.2, AC073046.1, CDKN2A−DT. The results of survival analysis, risk map, ROC curve, heatmap showed that the prognostic characteristics of the five LncRNAs associated with cuproptosis accurately distinguished high and low risk patients and reliably predicted the prognosis of UCEC patients as prognostic factors independent of other common clinical characteristics. Unfortunately, no studies have investigated the role of the above LncRNAs in tumorigenesis and progression. However, it also provides new ideas and directions for the follow-up study of endometrial cancer.

Endometrial cancer is usually divided into two types, type I is estrogen-dependent, and type II is non-estrogen-dependent (Setiawan et al., 2013). In recent years, some studies have found that there is crossover of molecular characteristics in this dualistic classification, and some cases are not completely consistent with pathological characteristics. Therefore, in 2013, TCGA divided endometrial cancer into four genomic subgroups. POLE hypermutation type; MSI-H type (microsatellite instability type) or mismatch repair system defect type (dMMR type); Low copy type (CNL), microsatellite stable type (MSS type) or non-specific molecular profile type (NSMP type); High copy type (CNH) or p53 mutation. Molecular typing of endometrial cancer is helpful to predict prognosis and guide treatment. The prognosis of POLE mutant is the best and patients with CNL have the worst prognosis. In this study, it was found that patients in the high-risk group were mostly concentrated in the CNH type, while patients in the low-risk group were mostly concentrated in the POLE mutant type, MSI type and CNL type (Kandoth et al., 2013). These results indicate that the evaluation of prognosis of endometrial cancer by Risk Score Model is basically consistent with that by TCGA molecular typing, which can better predict prognosis of patients with endometrial cancer, distinguish patients with high and low risk.

GO enrichment analysis showed that the molecular functions of LncRNAs related to cuproptosis were mainly antigen binding, extracellular matrix structural constituent. And the biological processes were mainly microtubule motility and cilia motility. KEGG enrichment analysis showed that LncRNAs were enriched in PI3K-AKT signaling pathway and MAPK signaling pathway. The PI3K-AKT signaling pathway is one of the most important intracellular pathways, and the main proteins involved in this signaling pathway are PI3K (phosphatidylinositol 3-kinase) and Akt (protein kinase B). This pathway is required for the induction of proliferation, growth, and differentiation of adult stem cells, especially neural stem cells (Adams et al., 2011). The PI3K-AKT signaling pathway is aberrantly activated in several cancers (Luwor et al., 2013). Currently, the two most common mechanisms of PI3K-AKT activation are triggered by somatic mutations in receptor tyrosine kinases (RTKS) and specific elements of the signaling pathway (Stommel et al., 2007). Although the exact etiology of endometrial cancer is still unclear, according to epidemiological investigation, high BMI, history of hypertension, history of metabolic syndrome, history of diabetes, history of hormone use and family inheritance are all high risk factors for endometrial cancer. Among them, the presence of excessive or unopposed estrogen in the body is an important risk factor (Lu and Broaddus, 2020). Studies have shown that after estrogen acts on human endometrial cells, the phosphorylation of PI3K is significantly increased, thereby inhibiting the apoptosis of endometrial cells, but the use of estrogen nuclear receptor antagonists cannot prevent this response, which indicates that as a causative agent, excessive or unopposed estrogen can activate the PI3K/AKT signal transduction pathway in endometrial cancer cells through non-transcriptional effects to promote cell proliferation (Qu et al., 2015). In addition, LncRNAs LINP1 and MEG3 have been shown to inhibit tumorigenesis and progression of UCEC cells through the PI3K/AKT pathway (Sun et al., 2017). Therefore, this pathway has become an important breakthrough point in the treatment of endometrial cancer, and many drugs that inhibit various components of this pathway are now in clinical trials. Experiments have shown that cisplatin may induce autophagy in endometrial cancer cells by inhibiting PI3K/AKT pathway, thereby exerting anti-tumor effect. Plumbagin (PLB), a naphthoquinone, has been shown experimentally to exert anticancer effects possibly by abolishing the PI3K/Akt pathway, suggesting that it is a promising phytotherapeutic candidate for EC therapy in the future (Zhang X. et al., 2021). Since endometrial cancer is associated with estrogen levels, endocrine therapy is also worthy of attention. Progesterone is commonly used as conservative endocrine therapy in young patients with early-stage endometrial adenocarcinoma. But according to clinical data, some of these patients do not respond to progesterone because primary or acquired progesterone resistance develops during progesterone therapy despite the use of different drugs or treatment regimens. Hua Liu et al. showed that activation of the PI3K/AKT pathway leads to the activation of mTOR and promotes cell proliferation by inhibiting autophagy. Inhibition of the PI3K/AKT/mTOR pathway reverses progesterone resistance in UCEC. That is to say, the effect of restoring the sensitivity of progesterone therapy in endometrial cancer cells is achieved (Liu et al., 2017). Irene et al. demonstrated that inhibition of the PI3K/AKT pathway reversed progesterone resistance by reducing non-genomic progestogen-mediated reactivation of the PI3K/AKT pathway, upregulated progesterone receptor subtype B transcriptional function and reduced angiogenesis in endometrial cancer (Lee et al., 2016). In addition, PI3K inhibitors niraparib and BAY 80-6946 are currently being tested in clinical trials to investigate their therapeutic effects on endometrial cancer, possibly by blocking some of the enzymes required for cell growth (Jiang et al., 2020).

When it comes to this pathway, PTEN gene has to be mentioned. The PTEN gene is the first tumor suppressor gene found to have lipid phosphatase and protein phosphatase bispecificant phosphatase activity. Its most important substrate is phosphatidylinositol (3, 4, 5) ⁃trisphosphate (PIP3), which is the product of the phosphatidylinositol⁃3’kinase (PI3K) and mediates AKT activation. PTEN can dephosphorylate PIP3 and maintain PIP3 at a low level, thereby down-regulating the PI3K/AKT pathway (Carnero et al., 2008). Studies have shown that mutation or deletion of PTEN can increase the proliferation, migration, invasion and cisplatin resistance of endometrial cancer cells (Chen et al., 2018; Wang et al., 2020). PTEN is the most frequently mutated gene in endometrial cancer and has the highest mutation rate in endometrioid adenocarcinomas, but mutations in serous carcinomas are very rare. Loss of PTEN function in the endometrium is considered to be an early event in carcinogenesis and is associated with a favorable prognosis, since PTEN mutation rates are higher in patients with endometrioid adenocarcinoma or precancerous lesions compared with patients with advanced or metastatic endometrial cancer. The waterfall plot of this study, which shows a higher PTEN mutation rate in the low-risk group, is a better indication of a higher PTEN mutation rate and a better prognosis.

The MAPK pathway is a cascade of three kinases, of which the upstream kinase (MAPKKK) responds to a variety of extracellular and intracellular signals and activates the intermediate kinase (MAPKK) through direct phosphorylation. MAPKK specifically phosphorylates and activates MAPK, which usually has many substrates and can carry out the corresponding commands. MAPK responds to a variety of inputs, including physiological signals such as hormones, cytokines, and growth factors, as well as endogenous stress and environmental signal (Lee et al., 2020). Among them, the Ras-Raf-MEK-ERK pathway plays an important role in the occurrence and development of cancer and targeted drug therapy. Studies have found that Asparanin A inhibits the migration and invasion of endometrial cancer cells through Ras/ERK/MAPK pathway (Zhang F. et al., 2021). However, some studies have found that LncRNA rhPN1-AS1 promotes the progression of endometrial cancer by activating the ERK/MAPK pathway. In terms of treatment, LncRNA HEIH can enhance the tolerance of endometrial cancer cells to paclitaxel by activating the MAPK signaling pathway (Guo et al., 2020).

Then, we analyzed the immune function, tumor mutation burden and others of UCEC patients. Heatmap of immune function analysis revealed differences in T-cell costimulatory and cytolytic activity between low and high risk groups. The costimulatory and cytolytic activities of T cells were inhibited in high-risk patients. Studies have shown that the combination of T cell costimulation and targeted FAK can enhance anti-tumor immunity, while the inhibition of T cell costimulation in high-risk population can weaken human anti-tumor immunity (Canel et al., 2020). Tumor mutation burden (TMB), which simply refers to the number of mutations within a tumor, has emerged as a useful biomarker for immune checkpoint blockade (ICB) in many cancer types to identify patients who would benefit from immunotherapy (Chan et al., 2019). Theoretically, the more mutations, the more neoantigens, and the higher the chance that one or more of these autoneoantigens will be immunogenic and trigger a T-cell response (Jardim et al., 2021). In our study, the tumor mutation burden was higher in the low-risk group, and the waterfall plot suggested that the low-risk group had a higher mutation rate for most genes. This also mean that patients in the low-risk group have more neoantigens and are more likely to be recognized by immune cells, and thus more likely to benefit from immunotherapy. Survival analysis showed that patients with high mutation burden had a better survival rate. Comprehensive survival analysis combined with risk score showed that patients with high mutation burden and low risk had the highest survival rate, and patients with low mutation burden and high risk had the lowest survival rate.

Of course, there are still limitations of this study. Only 548 UCEC cases and 41 healthy samples were included in this study, and the TCGA data were based on RNA-sequencing technology. Whether additional validation methods are needed is debatable. Second, the mechanism of cuproptosis-related LncRNAs in endometrial cancer warrants further investigation. Subsequent more detailed experiments should be performed to explore the exact mechanism. Through the analysis of drug sensitivity, we learned that patients in the high-risk group were more sensitive to Lapatinib, Paclitaxel, Pazopanib, Rapamycin, Sunitinib. These drugs are currently used in the treatment of breast cancer, ovarian cancer, lung cancer, renal cell carcinoma, melanoma, gastrointestinal stromal tumor, and the therapeutic mechanism of these drugs in UCEC patients needs to be further studied.

## 5 Conclusion

In this study, we identified 5 LncRNAs co-expressed with cuproptosis-related genes in UCECs, constructed a prognostic model of cuproptosis-related LncRNAs for UCECs, and analyzed the relationship between risk score-based grouping and tumor mutation burden and immunotherapy. Our study provides a new perspective for survival prediction and clinical treatment of UCEC patients.

## Data Availability

The datasets presented in this study can be found in online repositories. The names of the repository/repositories and accession number(s) can be found in the article/supplementary material.
